# Determining the ideal prevention strategy for multidrug-resistance organisms in resource-limited countries: a cost-effectiveness analysis study

**DOI:** 10.1017/S0950268820001120

**Published:** 2020-05-20

**Authors:** Ying Wang, Yufeng Yuan, Likai Lin, Xiaodong Tan, Yibin Tan

**Affiliations:** 1 Department of nosocomial infection management, Zhongnan hospital of Wuhan University, Wuhan, Hubei province, China; 2 Hospital Institute of Wuhan University, Wuhan, China; 3 Wuhan University School of Health Sciences, Wuhan, China

**Keywords:** Developing countries, economics, hand hygiene, intensive care unit, medical, microbial, patient isolation, resistance

## Abstract

The aim of this study was to determine the most cost-effective strategy for the prevention and control of multidrug-resistant organisms (MDROs) in intensive care units (ICUs) in areas with limited health resources. The study was conducted in 12 ICUs of four hospitals. The total cost for the prevention of MDROs and the secondary attack rate (SAR) of MDROs for each strategy were collected retrospectively from 2046 subjects from January to December 2017. The average cost-effectiveness ratio (CER), incremental cost-effectiveness ratio (ICER) and cost-effectiveness acceptability curve were calculated. Hand hygiene (HH) had the lowest total cost (2149.6 RMB) and SAR of MDROs (8.8%) while single-room isolation showed the highest cost (33 700.2 RMB) and contact isolation had the highest SAR of MDROs (31.8%). The average cost per unit infection prevention was 24 427.8 RMB, with the HH strategy followed by the environment disinfection strategy (CER = 21 314.67). HH had the highest iterative cost effect under willingness to pay less than 2000 RMB. Due to the low cost for repeatability and obvious effectiveness, we conclude that HH is the optimal strategy for MDROs infections in ICUs in developing countries. The cost-effectiveness of the four prevention strategies provides some reference for developing countries but multiple strategies remain to be examined.

## Introduction

The prevention and control of multidrug-resistant organisms (MDROs) is one of the most urgent public health concerns worldwide, especially in countries with limited health resources [1–3]. MDROs lead to a substantial economic burden due to unnecessary longer hospital stays, higher risk of readmissions and additional disease costs [4, 5]. Apart from the economic factors, the human disease burden is high, since mortality due to healthcare-associated infection (HAI) has been suggested to be 70% higher in patients with MDROs compared with those with antibiotic-susceptible infections [6].

Patients in the intensive care unit (ICU) are regarded as those at the highest risk of infection with MDROs due to invasive procedures, the use of immunosuppressive agents as well as a number of drugs (including antibiotics) and their underlying diseases [7, 8]. In China, a recent study showed that the detection rate of multidrug-resistant *Acinetobacter baumannii* (MDR-AB) in the ICU was 39.9%, compared with 13.4% in non-surgical departments [9], while in German ICUs, the prevalence of carbapenem-resistant organisms (CRO) was higher than in the general wards [10]. Direct contact with infected patients, carriers, medical equipment and the contaminated environment is believed to be the main route of transmission for MDROs in the ICU [11]. Hence, guidelines published by the World Health Organization (WHO) in 2017 proposed prevention and control measures for MDROs based on multimodal infection prevention and control (IPC) strategies [12]. Unfortunately, many hospitals in low and middle-income countries lack the necessary infrastructures, medical equipment and experienced professionals, leading to substantial challenges and dilemmas for preventing MDROs effectively [13]. A national health resource survey in China showed an unpromising status between the healthcare demands and poor support because the ratios of patient beds to doctors and nurses were 5:1 and 5:1.85, respectively and each ICU only had on average two single rooms available for isolation [14]. The WHO has identified the obvious gaps in understanding the cost-effectiveness and practicability in isolating patients with MDROs [12], in particular, how to maximise the effectiveness of prevention and control of MDROs in hospitals with limited resources especially in low- and middle-income countries and how to optimise the use of prevention and control resources.

Health economic analysis is a useful tool to evaluate the effect of various health policies and is being increasingly used in the analysis of the prevention and control measures of MDROs [15–17]. Previous studies have mainly focused on a single measure evaluation in developed countries [18–21], but did not consider the most cost-effective isolation measures with regard to limited health resources. Hospitals with low- and mid-level budgets and resources need to select and focus on an optimal strategy based on practical cost-effectiveness, rather than implementing a whole host of measures against MDROs.

Given the importance of the scientific and rationale prevention strategies for MDROs worldwide, the limited isolation resources in developing countries and the lack of related cost-effectiveness analysis researches, this study aimed to determine the ideal prevention strategy for MDROs in ICUs in areas with limited health resources, based on a decision tree model.

## Methods

### Study design and model construction

This retrospective study was conducted in 12 ICUs of four hospitals (three in each hospital) (Zhongnan Hospital of Wuhan University, Central Hospital of Wuhan, Union Hospital affiliated to Tongji Medical College of Huazhong University of Science and Technology and Tongji Hospital affiliated to Tongji Medical College of Huazhong University of Science and Technology) and covered the time period from January to December 2017. The four hospitals were large general tertiary grade A centres with 3300, 3398, 5000 and 6000 beds. Their ICUs are national large- and medium-sized and cover three key specialities in each hospital; ICU bed numbers were 59, 54, 55 and 65, respectively, and the 12 ICU wards were comparable in the number of beds and the condition of the admitted patients. The study was approved by the ethics committee of Zhongnan Hospital of Wuhan University. The authors assert that all procedures contributing to this work comply with the ethical standards of the relevant national and institutional committees on human experimentation and with the Helsinki Declaration of 1975, as revised in 2008.

The inclusion criteria of patients were: (1) complete basic information; and (2) patient consent to be included in the database and agreement on personal data collection. The four hospitals represented the four core infection prevention and control strategies, namely hand hygiene (HH), contact isolation, single room isolation and environmental surface cleaning and disinfection [12] performed according to each hospital's own guidelines.

### Hypothesis

We hypothesised that under different preventions, the number of patients infected with MDROs would vary in the ICUs over the observation period. At the same time, the cost of implementing prevention measures will vary according to the number of patients infected with MDROs in the ICU and the costs needed for prevention will increase with an increasing number of patients infected with such organisms. Therefore, under these assumptions, the secondary attack rate (SAR) of MDROs infection rates in each of the four hospitals (each applying one of the four strategies) were taken as the index of a prevention effect. The prevention cost of the four strategies multiplied by the number of infections was regarded as the accounting total cost. Based on patients' outcomes under the different prevention strategies and associated costs, an analytic decision model was built to evaluate the cost-effectiveness of different prevention strategies. This economic evaluation is in accordance with the WHO cost-effectiveness analysis guideline [22].

### Model data and input

There were two main data inputs in the decision model: the definition and calculation of the four prevention measures' cost and effectiveness. Since there are few comprehensive prospective studies on the dynamic transmission of MDROs in ICUs, especially in developing countries, we sought to retrospectively collect actual data from the study hospitals over 1 year. Based on the model construction, patients could be admitted to the ICU as being either infected, colonised, or uncolonised with MDROs.

Each prevention strategy had a focused key implementation and the cost per patient infected with MDROs was calculated accordingly (Table 1). Because of the limited information and published literature, we derived the detailed costs from three channels: the Official Price Bureau, hospitals' material supply system and direct observation as supported by screenshots or paper trail. In addition, two other kinds of costs during MDROs prevention were included, based on a previously published study [23] namely nursing time (12.8 Yuan/infected patient) and medical waste disposal (safe handling and transportation of medical waste bags once a day and transportation of sharps boxes every 2 days at 0.98 Yuan/infected patient). The total cost of each strategy was calculated by multiplying the cost per unit infected patient by the number of infected patients in the ward during the study period.

**Table 1.** Cost definition and calculation for MDRO prevention and control measures in the model

Strategy	Definition	Reference	Cost (RMB/infected patient)^a^
Single-room isolation	Separation of patients in MDRO wards and a series of other infection control measures. Patients received treatments in a single room; attended by specialist physicians and arrangement for specialised equipment for treatment.	Price Bureau	158
Contact isolation	Contact isolation measures included bedside availability of disposable clothing, gloves, caps, shoe covers, etc. And with strict implementation of isolation-related procedures for patients, family and staff.	Material system	236.6
Hand hygiene	Access of medical staff having access to rapid-acting hand sanitizers, access to mobile devices and completion of HH self-assessments on mobile devices after high-risk procedures. Installation of cameras near sinks to monitor HH practice and to arrange managers to supervise procedures.	Direct observation; Material system	27.0864
Environmental surface cleaning and disinfection	Importance of disinfection of patient's surrounding environment. Frequency of intensive cleaning and disinfection of instruments and objects used by the patient, wiping down surfaces with chlorine-containing disinfectants (500 mg/l) or disinfection with wet paper towels more than three times a day and disinfection of bed units with ozone once a week.	Direct observation; Material system	82.248

MDROs, multidrug-resistant organisms; HH, hand hygiene.

a Costs were calculated on a per-patient basis. Total cost over the study period was determined by individual patient's cost times the number of infected patients in the ward.

We postulated that under the different prevention strategies implemented in each hospital, the patients' infection status and the rate of new infections in the wards would be different. The effectiveness of this evaluation was regarded as the SAR of MDROs in the wards. The reverse calculation was used as the standard in the statistical analysis. SAR is an effective index widely used in epidemiology and infection for evaluating the prevention and control measures of infectious diseases [24]. A retrospective cohort study used SAR to assess the effect of different prevention measures on Middle-East respiratory syndrome [25]. Similarly, SAR was used as the major index to assess the effectiveness of antiviral prophylaxis during the HIN1 outbreak [26]. The calculation and estimation of the number of cases were derived from the retrospective data and based on the following formula [24]:
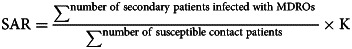


### Outcomes and definitions

The key indicators in this study were: (1) cost-effective frontier (CEF): in the cost-effect diagram, the cost for all strategies are connected by line segments to form a cost effect boundary for cases of multiple strategies and absence of inferior strategies; (2) average cost-effectiveness ratio (CER): average cost per unit infection prevented; (3) incremental cost-effectiveness ratio (ICER): represents the cost of unit effectiveness (ΔC/ΔE). The effectiveness of strategies was based on SAR and ICER referred to the cost to prevent one new secondary infected patient in the ward; (4) a tornado analysis was performed to determine the value that would change the choice of the optimal decision within the change range of uncertain variables; (5) cost-effectiveness acceptability curve (CEAC): the maximum additional cost that policymakers were willing to pay for a unit effect (WTP) was set at 2000 RMB, as determined by practical experience; through the simulation analysis in different ranges of the WTP values (*λ*), the scatter plot (*λ*-*δ*) and CEAC can be drawn; and (6) net monetary benefits (NMB): NMB = E × WTP – C; the NMB considers the factors from the cost, effectiveness and WTP.

### Statistical analysis

The statistical significance of the basic patients' characteristics among the four strategies was determined using *χ*^2^ tests and ANOVA with Tukey's post hoc test, using the STATA software (StataCorp LP, College Station, TX, USA). *P* values <0.05 denoted statistical significance. For the cost-effectiveness analysis, the relevant parameters were entered into Excel (Microsoft, Redmond, WA, USA) and analysed using TreeAge 11.0 (TreeAge Software, Inc., Williamstown, MA, USA).

## Results

### Characteristics of the patients

A total of 2046 patients from the 12 ICUs were included in the study. The numbers of patients in each strategy were 587 with HH, 409 with single-room isolation, 444 with contact isolation and 606 with surface cleaning and disinfection. There were significant differences among the four groups regarding age, ICU stay, surgery, type of sample for bacterial detection, numbers of routine or abnormal tests and use/duration of airway ventilation, central and urinary catheters (all *P* < 0.00; Table 2). The SAR of MDROs was highest with the contact isolation strategy (31.8%) and the lowest with HH strategy (8.8%), single-room isolation (15.9%) and environmental disinfection (24.6%) falling in between (Table 2).

**Table 2.** Demographic and clinical characteristics of the patients

Category	Hand hygiene	Single-room isolation	Contact isolation	Environment disinfection	*P*
	(*n* = 587)	(*n* = 409)	(*n* = 444)	(*n* = 606)	
Gender					0.246
Male	337 (57.4%)	241 (58.9%)	268 (60.4%)	374 (61.7%)	
Female	250 (42.6%)	168 (41.1%)	176 (39.6%)	232 (38.3%)	
Age (years)	66.2 ± 15.8	67.0 ± 19.9	66.1 ± 17.7	65.7 ± 17.7^a^^b^^c^	<0.001
Intensive care unit stay (day)	17.4 ± 22.4^a^	15.9 ± 24.2^a^	24.9 ± 29.9^a^^b^	18.9 ± 20.1^c^	<0.001
Surgery					<0.001
Yes	431 (73.4%)	314 (76.8%)	375 (84.5%)	541 (89.3%)	
No	156 (26.6%)	95 (23.2%)	69 (15.5%)	65 (10.7%)	
Sample type used for bacterial detection					<0.001
Sputum	248 (42.2%)	146 (35.7%)	156 (35.1%)	129 (21.3%)	
Blood	68 (11.6%)	57 (13.9%)	69 (15.5%)	70 (11.6%)	
Urine	49 (8.4%)	37 (9.1%)	43 (9.7%)	50 (8.3%)	
Chest and abdomen drainage fluid	43 (7.3%)	22 (5.4%)	35 (7.9%)	47 (7.6%)	
Non	179 (30.5)	147 (35.9%)	141 (31.8%)	310 (51.2%)	
Number of routine blood tests	42.2 ± 51.8	42.6 ± 41.3	54.5 ± 47.157	57.1 ± 46.4	0.18
Numbers of abnormal routine blood tests	27.5 ± 30.5	25.6 ± 26.1	34.1 ± 35.2^a^^b^	31.4 ± 33.6^a^^b^	<0.001
Number of routine urine test	4.0 ± 3.8	4.8 ± 5.4	6.6 ± 7.0^a^^b^	12.5 ± 13.1^a^^b^^c^	<0.001
Numbers of abnormal routine urine tests	1.9 ± 2.9	2.2 ± 3.6	3.8 ± 5.4^a^^b^	7.0 ± 9.5^a^^b^^c^	<0.001
Number of procalcitonin tests	4.2 ± 3.8	5.2 ± 4.2^a^	8.9 ± 10.0^a^^b^	9.6 ± 9.7^a^^b^	<0.001
Numbers of abnormal procalcitonin	3.6 ± 3.6	4,6 ± 4.1	6.6 ± 7.0^a^^b^	12.5 ± 13.1^a^^b^	<0.001
Number of patients with ventilator use	0.2 ± 0.6	1.3 ± 1.3^a^	2.0 ± 1.8^a^^b^	2.0 ± 1.73^a^^b^	<0.001
Ventilator use (days)	2.2 ± 13.6	7.0 ± 10.9^a^	16.4 ± 30.9^a^^b^	13.0 ± 20.0^a^^b^^c^	<0.001
Number of patients with central venous catheterisation use	0.1 ± 0.5	1.5 ± 1.6^a^	2.6 ± 2.2^a^^b^	2.7 ± 2.2^a^^b^	<0.001
Central venous catheterisation use (days)	1.6 ± 7.3	16.8 ± 23.1^a^	25.5 ± 27.8^a^^b^	22.4 ± 29.7^a^^b^	<0.001
Number of patients with urinary catheter	0.2 ± 0.7	1.6 ± 1.8^a^	2.5 ± 2.0^a^^b^	2.9 ± 1.9^a^^b^^c^	<0.001
Urinary catheter (days)	4.1 ± 18.0	16.7 ± 23.3^a^	27.5 ± 34.9^a^^b^	26.0 ± 32.3^a^^b^	<0.001
SAR of MDROs	8.8%	15.9%	31.8%	24.6%	<0.001

SAR, secondary attack rate; MDROs, multidrug-resistant organisms.

a *P* < 0.05 compared to hand hygiene.

b *P* < 0.05 compared to single-room isolation.

c *P* < 0.05 compared to contact isolation.

Table 3 lists the multidrug-resistant microorganisms recovered from patients and shows that the most frequent were carbapenem-resistant *Enterobacteriaceae*, followed by carbapenem-resistant *Acinetobacter baumannii* and methicillin-resistant *Staphylococcus aureus*.

**Table 3.** Isolation of multidrug-resistant microorganisms over the study period

MDROs	Hand hygiene (*n* = 587)	Single-room (*n* = 409)	Contact isolation (*n* = 444)	Environment disinfection (*n* = 606)
CRE	299 (50.9%)	237 (57.9%)	257 (57.9%)	399 (65.8%)
CRAB	247 (42.1%)	155 (37.9%)	208 (46.8%)	199 (32.8%)
VRE	35 (6.0%)	28 (6.8%)	36 (8.1%)	12 (2.0%)
MRSA	75 (12.8%)	49 (12.0%)	57 (12.8%)	66 (10.9%)
ESBL-producing *E. coli*	35 (6.0%)	8 (2.0%)	0	36 (5.9%)
CRPA	8 (1.4%)	4 (1.0%)	5 (1.1%)	6 (1.0%)

MDROs, multidrug-resistant organisms; CRE, carbapenem-resistant *Enterobacteriaceae*; BRAB, carbapenem-resistant *Acinetobacter baumanii*; VRE, vancomycin-resistant *Enterococcus*; MRSA, methicillin-resistant *Staphylococcus aureus*; ESBL, extended-spectrum beta-lactamase; CRPA, carbapenem-resistant *Pseudomonas aeruginosa*.

*Note*: Some patients with multiple infections

### Cost-effectiveness analysis of the four strategies

**Fig. 1.**
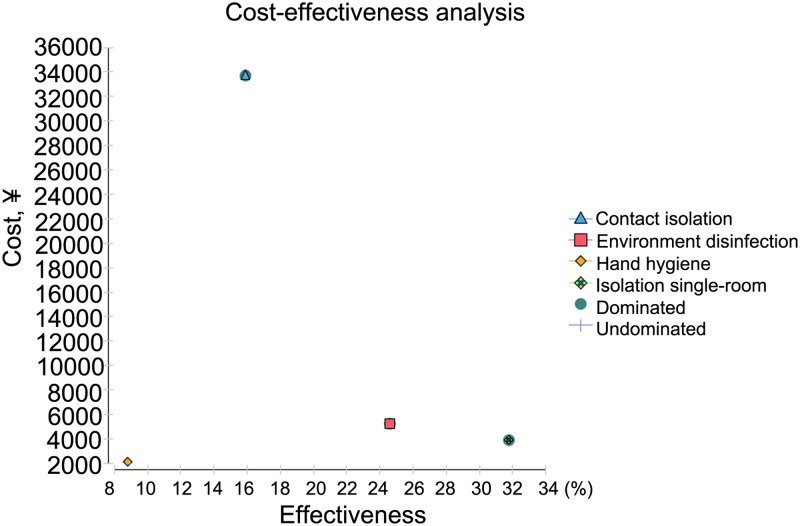
Cost-effectiveness analysis of the four intervention strategies.

Among the four major prevention measures, only HH and contact isolation were visible on the cost-effectiveness curve, while isolation in single-rooms and environmental disinfection fell beyond the cost-effectiveness curve (Fig. 1). HH had the lowest total cost (2149.6 RMB) and single-room isolation had the highest cost (33 700.2 RMB). The average cost per unit infection prevention was 24 427.8 RMB, with the HH strategy followed by environmental disinfection (CER = 21 314.67). Incremental costs for each additional unit of infection prevented were 0 and – 19 589.80 for the HH and environment disinfection strategies, respectively (Table 4). The tornado analysis showed that the number of new patients infected with MDROs under the HH strategy was the only factor that had the largest impact on the net benefit of the overall strategy.

**Table 4.** Cost-effective analysis of four infection control strategies

Strategy	Ranking	Cost	Effectiveness	CER^a^	incrCost	incrEff	ICER^b^	NMB
Hand hygiene	Undominated	2149.6	0.088	24 427.8	0	0	0	389.6
Environment disinfection	Abs. dominated	5243.4	0.246	21 314.7	3093.8	−0.158	−19 580.8	323.4
Single-room isolation	Abs. dominated	3901.9	0.159	12 277.8	1752.2	−0.2298	−7625.0	2454.1
Contact isolation	Abs. dominated	33 700.2	0.3178	211 950.7	31 550.5	−0.0071	−444 373.6	30 520.2

CER, cost-effective frontier; incrCost, incremental cost; incrEff, incremental effectiveness; ICER, incremental cost-effectiveness ratio; NMB, net monetary benefits; Abs. dominated, absolute dominated (i.e., when there are multiple strategies to choose from, there is always a strategy that is better than an absolute disadvantaged strategy); Undominated (i.e., in the cost-benefit analysis, an undominated strategy indicates that when there are multiple strategies to choose from, the dominant strategy is better than the others).

a average cost-effectiveness ratio.

b incremental cost-effectiveness ratio.

### CEAC for WTP

**Fig. 2.**
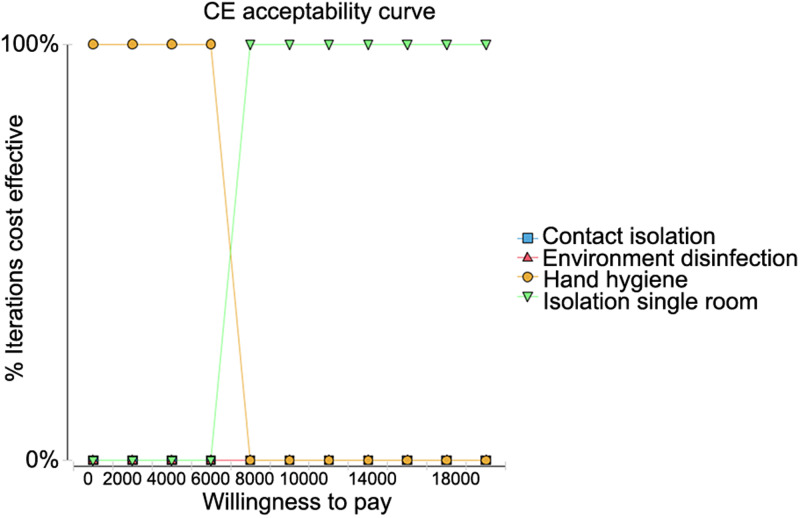
Cost-effectiveness acceptability curve when the willingness to pay (WTP) was set to 2000 RMB.

The cost-effectiveness acceptance curves for the four strategies were compared when the WTP for measures to prevent the transmission of MDROs was set at 2000 RMB. As shown in Figure 2, HH had the highest iterative cost effect under willingness to pay less than 2000 RMB meaning that it can be repeated at a very low cost. When the WTP was increased to 8000 RMB, single room isolation became the best strategy.

## Discussion

The cost-effectiveness analysis of four prevention strategies against MDROs in ICUs was studied in China, a country with limited health resources. HH proved to be the optimal infection prevention strategy due to its excellent performance and lower costs for each additional unit of SAR prevented, followed by contact isolation. The net benefit of the overall infection prevention strategy was mostly due to the effectiveness of the HH strategy which due to its low cost (<2000 RMB) for repetitive operation, would be more likely to be adopted by health care providers at the national or local level, for the prevention of MDROs.

While HH practice can readily be adopted by all healthcare staff, other strategies, in particular, environmental cleaning often requires dedicated staff members and specialist equipment. Likewise, single-room isolation requires that wards are designed to include such rooms, which is not the case in most hospitals in China since most wards only have one or two single rooms [14]. As expected, contact isolation proved the most expensive and impractical infection control strategy for developing countries because of the need for expendables and single-use supplies. It follows that the ideal setting, like in most hospitals in developed countries, is the simultaneous use of all four modalities [12], but the key finding from the present study is that HH alone was the most efficient of all approaches for preventing MDROs due to the direct reduction of SAR of MDROs, combined with the lowest cost. Nevertheless, human factors will ultimately determine the efficacy of these methods and the transmission of MDROs in hospitals [27].

The WHO-5 campaign has been shown to be effective in improving HH [28, 29], but in low- and middle-income countries, poor HH compliance of <10% remains typical [30, 31]. The benefit of HH alone is supported by a number of studies, as shown by a meta-analysis of the relation between HH and the incidence rate reduction of nosocomial infections in ICUs [32]. Moreover, a recent study by Luangasanatip *et al*. [33] showed that HH interventions are cost-effective in preventing MRSA in ICUs in middle-income countries and a study from Vietnam reported a saving of $1074 per prevented HAI through improved HH [34]. Individualised bundles of infection control measures, including HH, were also identified as a recommended strategy (ICER = $ 20 444.6) in the latter study. Due to its simplicity, attainability and economic benefit, our study underlines the effectiveness of HH for preventing MDROs transmission in hospitals and additionally can be extended to areas with limited health resources [28]. It is noteworthy that the effectiveness of HH depends on the compliance of healthcare workers. A study of >140 000 HH opportunities monitored by 4000 unique observers showed that an improvement of HH compliance from 80% to 95% could decrease the HAI rate and result in savings of about $5 million [35]. Hence, for HH, the compliance-dependent effect is particularly important when the cost fluctuation is small. For HH, the monetary investment is small, but the investment in time is more important, which is a primary factor playing against the strategy [36]. Insisting on better practice, using surveillance and alarms and providing feedback to staff could all contribute to improving the HH rates [36, 37].

Contamination of the near-patient environment by MDROs was found to be responsible for patient-to-patient transmission of these organisms [38–42]. Nevertheless, as for HH, compliance with the best principles of surface and environmental cleaning was found to be relatively low, with an average of 38% [43–45]. In addition, the significant material and human resources are needed for optimal environment control and have been shown to be less cost-efficient than HH [46].

Single-room isolation is one of the oldest methods for infection control, but it is time-consuming, may impede proper care and is inefficient if a total contact isolation strategy is used since the health care staff may spread infections [47]. The contact isolation strategy, especially for MSRA, remains controversial. Spence *et al*. [48] concluded that contact isolation was costly and unnecessary for patients colonised with MRSA, but an evaluation of 46 independent studies found there was some evidence supporting the practice [49]. Considering the low compliance level of HH in some developing countries [30, 31], single-room isolation or other sequential isolation measures could prove easier to implement than routine HH.

Some previous studies have explored the cost-effectiveness of prevention measures for HAIs, including MDROs, using a mathematical model; a systematic review documented that most of the cost-effectiveness analyses of control measures for MRSA remained at the level of individual measures, with very few studies comparing multiple measures [50]. Rattanaumpawan *et al*. [5] compared a combination of measures with traditional infection control care but did not analyse the individual components of the combination. Moreover, up to 2012, most of the economic evaluations of prevention measures against MRSA were carried out in developed countries such as the USA, Germany and Commonwealth countries [8, 10, 16, 18, 20, 21], with important gaps in knowledge regarding developing countries. The latter gaps combined with other shortages in health resources together contribute to hinder the prevention and control of MDRO transmission in such countries. Effective strategies are necessary to slow down the epidemics of MDROs, which potentially will have disastrous impacts on public health in the future [12, 51].

The present study has some limitations. First, while it investigated the transmission data of patients infected with MDROs and a decision-making model was developed, we recognise that the development of infectious diseases is an event-dependent process. Second, costs were calculated according to each patient with the total cost over during the study (expenditure per patient × number of infected patients in the ward), it did not take into account the patient's hospitalisation time and other factors. Hence, for future studies, we intend to establish a complete Markov model, including a time model based on the complete data of the outcomes of patients infected with MDROs in ICUs. Based on this, we will include the incremental cost that relies on the transition period of patients to perform in-depth cost-benefit analysis [52]. Nevertheless, we suggest that our study was innovative in determining the cost-benefit analysis of the different measures against MDROs in ICUs and constitutes an exemplar for developing countries. Third, each hospital relied on a different strategy for preventing MDROs and thus some bias could have resulted from differences between hospitals that were not taken into account in the study. Finally, multivariable analysis of the use of devices was not performed owing to the wide variability of the number of devices and timing of use among the patients. Such shortcomings need to be addressed in further studies to determine the optimal MDRO prevention strategy in different settings.

In summary, to our knowledge, this is the first study to evaluate the optimal strategy among HH, single-room isolation, contact isolation and environmental sanitation in developing countries for the prevention of MDROs. Due to the low cost for repeatability and obvious effectiveness, HH represents the optimal strategy to reduce the incidence of MDROs infection in ICUs in limited health resources and our findings may be relevant to low- and middle-income areas. Further research is needed on how to maximise the effectiveness of HH and the optimum combination of different prevention measures.
